# O-GlcNAc transferase regulates collagen deposition and fibrosis resolution in idiopathic pulmonary fibrosis

**DOI:** 10.3389/fimmu.2024.1387197

**Published:** 2024-04-11

**Authors:** Shia Vang, Eric Scott Helton, Yiming Guo, Bailey Burpee, Elex Rose, Molly Easter, Seth Bollenbecker, Meghan June Hirsch, Emma Lea Matthews, Luke Isaac Jones, Patrick Henry Howze, Vasanthi Rajasekaran, Rebecca Denson, Phillip Cochran, Isaac Kwame Attah, Heather Olson, Geremy Clair, Girish Melkani, Stefanie Krick, Jarrod Wesley Barnes

**Affiliations:** ^1^ Department of Medicine, Division of Pulmonary, Allergy and Critical Care Medicine, Heersink School of Medicine, University of Alabama at Birmingham, Birmingham, AL, United States; ^2^ Department of Pathology, Division of Molecular and Cellular Pathology, Heersink School of Medicine, University of Alabama at Birmingham, Birmingham, AL, United States; ^3^ Biological Science Division, Pacific Northwest National Laboratory, Richland, WA, United States

**Keywords:** OGT, O-GlcNAc, IPF, fibrosis, collagen, Smad3

## Abstract

**Background:**

Idiopathic pulmonary fibrosis (IPF) is a chronic pulmonary disease that is characterized by an excessive accumulation of extracellular matrix (ECM) proteins (e.g. collagens) in the parenchyma, which ultimately leads to respiratory failure and death. While current therapies exist to slow the progression, no therapies are available to resolve fibrosis.

**Methods:**

We characterized the O-linked N-Acetylglucosamine (O-GlcNAc) transferase (OGT)/O-GlcNAc axis in IPF using single-cell RNA-sequencing (scRNA-seq) data and human lung sections and isolated fibroblasts from IPF and non-IPF donors. The underlying mechanism(s) of IPF were further investigated using multiple experimental models to modulate collagen expression and accumulation by genetically and pharmacologically targeting OGT. Furthermore, we hone in on the transforming growth factor-beta (TGF-β) effector molecule, Smad3, by co-expressing it with OGT to determine if it is modified and its subsequent effect on Smad3 activation.

**Results:**

We found that OGT and O-GlcNAc levels are upregulated in patients with IPF compared to non-IPF. We report that the OGT regulates collagen deposition and fibrosis resolution, which is an evolutionarily conserved process demonstrated across multiple species. Co-expression of OGT and Smad3 showed that Smad3 is O-GlcNAc modified. Blocking OGT activity resulted in decreased phosphorylation at Ser-423/425 of Smad3 attenuating the effects of TGF-β1 induced collagen expression/deposition.

**Conclusion:**

OGT inhibition or knockdown successfully blocked and reversed collagen expression and accumulation, respectively. Smad3 is discovered to be a substrate of OGT and its O-GlcNAc modification(s) directly affects its phosphorylation state. These data identify OGT as a potential target in pulmonary fibrosis resolution, as well as other diseases that might have aberrant ECM/collagen accumulation.

## Introduction

Idiopathic pulmonary fibrosis (IPF) is a chronic lung disease and is characterized by an excessive accumulation of extracellular matrix (ECM) proteins, including type I and type III collagens and fibronectin, in the parenchyma that ultimately leads to a stiff microenvironment, respiratory failure and death (1, 2). Currently, there is no cure for IPF and prognosis remains poor with a median survival of less than 5 years after initial diagnosis (3–5). FDA approved pharmacologic treatments (Pirfenidone/Nintedanib) exist and slow disease progression by reducing fibroblast proliferation, collagen synthesis, and attenuating fibrogenic growth factor signaling (6). One of the major growth factor targets is transforming growth factor-beta (TGF-β), which induces collagen expression and accumulation via the canonical Smad3 pathway (4, 7–11). Interestingly, only a small portion of patients respond to the current therapies and many patients experience challenging side effects (4, 12). Most importantly, these treatments do not resolve the fibrotic process itself. Thus, identification of new molecular targets that are more comprehensive and focused on both preventing progression and resolving fibrosis are needed.

Key pathologic cell types in IPF include alveolar epithelial cells, macrophages, endothelial, and mesenchymal cells (fibroblasts and myofibroblasts) (13). It is well established that myofibroblasts are the ultimate effector cells in IPF, which are characterized by abundant expression and deposition of collagens (types I, III, IV, V, and VI) and fibronectin into the ECM (14). Cellular differentiation into mesenchymal cells [epithelial-mesenchymal transition (EMT) and endothelial-mesenchymal transition (EndMT)] and the fibroblast-to-myofibroblast transition (FMT), important features of fibrosis, are driven by TGF-β1/Smad3 signaling and mechanostimulation through ECM remodeling which is regulated by activation/suppression of matrix metalloproteases (MMPs) or collagenases (15–20). Excessive secretion and remodeling of ECM proteins are high-energy processes, and this metabolic adaptation may hold critical information to understanding the changes that occur in myofibroblast biology and fibrosis resolution (21, 22).

**Figure 1:**
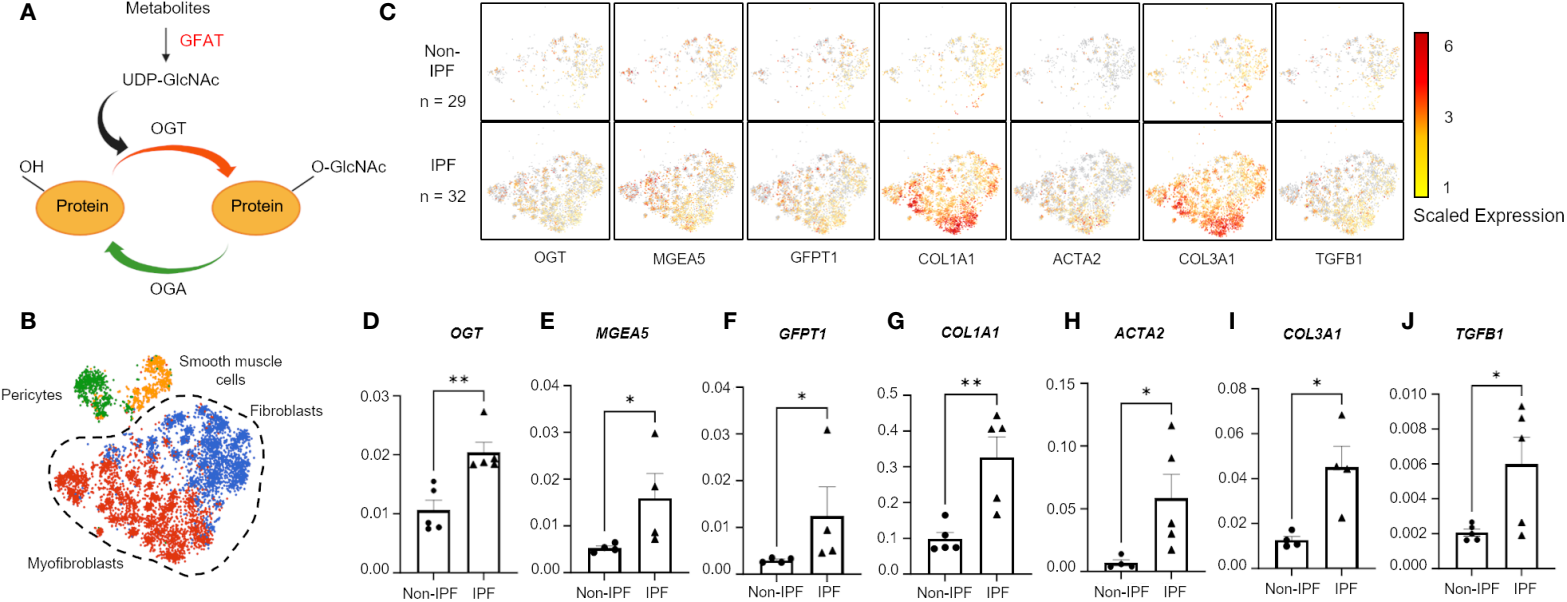
Hexosamine biosynthetic pathway genes are upregulated in IPF fibroblasts and myofibroblasts. **(A)** Schematic of simplified HBP and OGT/OGA cycling of O-GlcNAc. Image created with Biorender. **(B)** Cluster map of cell types: pericytes, smooth muscle cells, fibroblasts, and myofibroblasts, expressing *CTHRC1* from IPF (n=32) and non-IPF (n=29) donors. **(C)** Spatial mapping of fibroblasts and myofibroblasts expressing HBP and profibrotic genes based on scaled expression, gray coloration denotes no expression. **(D–J)** Transcript expression of HBP genes and profibrotic genes from 5 IPF donors and 5 non-IPF primary human lung fibroblasts. Data from multiple replicates are presented as mean ± SEM, each dot represents biological replicates averaged from three technical replicates. Outliers determined by the 1.5xIQR rule were excluded, and statistical analyses were done using the Student’s t-test. *p<0.05, **p<0.01. GFAT, Glutamine fructose-6-phosphate aminotransferase; *GFPT1*, glutamine fructose-6-phosphate transaminase 1; O-GlcNAc, O-linked N-Acetylglucosamine; OGT, O-GlcNAc transferase; OGA, O-GlcNAc hydrolase; *MGEA5*, meningioma expressed antigen 5; *ACTA2*, actin, alpha 2; smooth muscle.

The extensive energy requirements of ECM remodeling often require excess metabolites from different metabolic pathways, including glucose, glutamine, and acetyl-CoA (23, 24). A portion of these metabolites are funneled into the hexosamine biosynthetic pathway (HBP) (**Figure 1A**) (25). The HBP is critical for the production of the nucleotide sugar, uridine diphosphate N-Acetylglucosamine (UDP-GlcNAc), which is an essential substrate for almost all oligosaccharides that make up glycoconjugates (25, 26). UDP-GlcNAc is enzymatically converted into the O-linked GlcNAc (O-GlcNAc) modification via O-GlcNAc transferase (OGT). O-GlcNAc is a single monosaccharide addition to proteins at unoccupied serine and threonine residues, analogous to protein phosphorylation (27, 28). O-GlcNAc removal is mediated by OGA (O-GlcNAc hydrolase) and this GlcNAc cycling between OGT and OGA is a dynamic process under the influence of the cellular microenvironment (i.e., nutrient flux and stress) (26, 29). For this reason, the O-GlcNAc modification has been well documented as a metabolic sensor that regulates many cellular functions (26, 27, 29, 30).

The O-GlcNAc modification has been shown to tightly control proteins involved in transcription/translation, cell cycle, signal transduction, and protein processing and has been studied in many diseases such as cancer, Alzheimer’s disease, heart failure, pulmonary hypertension and diabetes mellitus (27, 30–33). Sustained increases or decreases in O-GlcNAc levels are pathological, indicating that a homeostatic level of O-GlcNAc is crucial for normal cell function (31, 32, 34–36). Evidently, both increased and decreased O-GlcNAc levels have been associated with liver and renal fibrosis, respectively (34, 35). However, investigations into specific molecular mechanism(s) that involve the O-GlcNAc modification in collagen deposition and/or fibrosis resolution have not been fully elucidated.

In this report, we showed that OGT regulates collagen deposition and resolution, a process that we demonstrated is evolutionarily conserved across multiple species. We identified multiple O-GlcNAc modified proteins in human lung fibroblasts that might directly regulate collagen in fibrosis/resolution, including the TGF-β effector molecule Smad3. Molecular insight into the regulation of Smad3 reveals that the O-GlcNAc modification controls Ser-423/425 phosphorylation, and subsequently regulates collagen expression/deposition. The role of OGT as a potential target in pulmonary fibrogenesis and fibrosis resolution, as well as other diseases with collagen accumulation or impaired wound repair is discussed.

## Materials and methods

### IMR-90 human lung fibroblast cell line

IMR-90 normal human lung fibroblasts, a commercially available primary line of lung fibroblasts isolated from a clinically normal 16-week female fetus (cat no: I90-83; RRID : CVCL_0347), were purchased from Coriell Institute. Fibroblasts at passage numbers 5-9 were used in this study. Cell authentication of fibroblasts lines were verified by morphology and examination of type I collagen and (α-SMA) as described previously (37) by our lab and Coriell Institute. Fibroblasts were cultured in DMEM (cat no: 15-013-CM; Corning) supplemented with 10% fetal bovine serum (FBS) (cat no: FP-0500-A; Atlas), 1X Penicillin-Streptomycin (cat no: 30-002-CI; Corning), and 1X GlutaMAX (cat no: 35050-061; Thermo Fisher Scientific) in a CO_2_ (5%) incubator at 37°C.

### Primary human lung fibroblasts

Primary human IPF and non-IPF fibroblasts/tissues were obtained from University of Alabama at Birmingham (UAB) Tissue Procurement Facility, derived from de-identified lung tissues, which protocol was approved by the UAB Institutional Review Board and conformed to the Declaration of Helsinki. The diagnosis of IPF was made by a multidisciplinary approach according to American Thoracic Society/European Respiratory Society guidelines (38). Primary HLFs were cultured following the same protocol as IMR-90s.

### Mouse studies

Aged 18-month-old (72 weeks old or greater) C57BL/6J (RRID#IMSR_JAX:000664) male mice were obtained from the Jackson Laboratories (Bar Harbor, Maine, USA). Male mice were used for the study since they develop a more severe fibrotic response from a single-dose of bleomycin compared to female mice (39). Animals were housed under pathogen-free conditions with food and water ad libitum. Littermates of the same sex were randomly assigned to experimental groups. All experiments and procedures were approved by the Institutional Animal Care and Use Committee at the University of Alabama at Birmingham (Birmingham, AL).

### Drosophila studies

*Drosophila melanogaster* stocks were raised with a standard regular diet: agar 11 g/L, active dry yeast 30 g/L, yellow cornmeal 55g/L, molasses 72mL/L, 10% nipagen (in ethanol), propionic acid 6mL/L. To evaluate OGT regulation of pericardin, transgenic stocks were obtained from Bloomington Drosophila Stock Center (BDSC) and Vienna Drosophila Resource Center (VDRC): *w^1118^* (BDSC: 3605), *UAS-Sxc* (BDSC: 83725), *UAS-Sxc*-RNAi (BDSC: 82460), control RNAi (BDSC: 36303), and *UAS-GFP* (BDSC: 5431), *r4-Gal4* (BDSC: 33832), and *Ubi-Gal4* (BDSC: 32551). *Act88F-Gal4* was obtained from the laboratory of Richard Cripps (40). Flies were housed at 25 °C, 50% humidity in a 12 h light/12 h dark cycle. Flies were transferred onto fresh media every 3 days.

### Bacteria and culture

*E. coli* strain DH5α (cat no: C2987H; New England Biolabs), Turbo (cat no: C2984H; New England Biolabs) and BL21 (DE3) (cat no: C2527H; New England Biolabs) were used for cloning and protein expression, respectively. Wild-type *E. coli* isolates were grown at 37°C in Luria Broth (LB) (cat no: IB49020; IBI Scientific) medium. Engineered strains with pET-Duet-hOGT-hSMAD3 were grown in LB plus ampicillin (100 mg/mL) at 37°C. Protein expression was induced with 0.2 mM isopropyl β-D-1thiogalactopyranoside (IPTG) (cat no: 34060; Thermo Fisher Scientific) overnight at 25°C in a shaker rotating at 250 rpm.

#### Mining from scRNA-seq dataset

The publicly available single-cell RNA sequencing dataset GSE147066 (41) was used to generate spatial/expression maps of markers for the FMT and genes involved in the HBP. Cluster maps were created using BBrowser3 (Bio Turing Inc, San Diego, CA, USA). A total of 1,144 non-IPF cells and 5,463 IPF cells were obtained from 29 non-IPF and 32 IPF donors, respectively. Expression maps were generated using BBrowser3.

#### Cell culture

Primary non-IPF and IPF HLFs obtained from the UAB Tissue Procurement Facility were seeded in 6-well plates (cat no: 353046, Corning) at an average density of 3 x 10^4^ and grown until cells reached 70-80% confluency before harvesting.

For 48 hour assays, HLFs were seeded in 6-well plates at an average density of 4 x 10^4^ cells per well. After overnight seeding, cells were serum starved with 0% FBS the following day for 16 hours. Following serum starvation, cells were pre-treated with OSMI-1 (25 μM) (cat no: SML1621, Millipore Sigma), OSMI-4 (10 μM) (cat no: HY-114361, Medchem Express), or TMG (1 μM) (cat no: 13237, Cayman Chemical) for 2 hours in normal media followed by TGF-β1 (5 ng/mL) (Cat no: 100-21, Peprotech) addition. Plates were harvested after 48 hours with TGF-β1 stimulation.

For 2 hour assays, HLFs were seeded in 12-well plates (Cat no: 353043, Corning) at an average density of 5 x 10^4^ cells per well. After overnight seeding, cells were serum starved with 0% FBS the following day for 16 hours. Following serum starvation, cell were pre-treated with OSMI-1 (25 μM) or TMG (1 μM) for 2 hours in serum starved media followed by a time-dependent TGF-β1 (5 ng/mL) stimulation for up to 2 hours. Plates were harvested after 2 hour time course.

#### RNA isolation, cDNA synthesis and quantitative PCR

Total RNA extraction (human lung fibroblasts, cat no: K0732; Thermo Fisher Scientific; mice lung homogenate, cat no: IB47302; IBI Scientific), cDNA synthesis (cat no: K1652; Thermo Fisher Scientific), RT-qPCR, and gene expression analysis were performed as previously described (42, 43). Briefly, total RNA extraction from HLFs or mice lung homogenate was performed according to the manufacturer’s protocol. Isolated RNA was quantified using a microplate reader (Biotek Synergy Mx) and 1 ug of cDNA was synthesized following manufacturer’s protocol using a thermocycler (PCT-100; MJ Research). The PCR conditions were cycled as followed: 25°C for 10 minutes (step 1), 50°C for 15 minutes (step 2), and 85°C for 5 minutes (step 3). For gene expression analysis, 10 ng of cDNA was used for quantitative PCR (qPCR) (StepOnePlus Real-Time PCR; Thermo Fisher Scientific) with the following Taqman probes (Life Technologies/Applied Biosystems): GAPDH (Hs02758991; Mm99999915_g1), OGT (Hs00269228_m1; Mm00507317_m1), MGEA5 (Hs00201970_m1; Mm00452409_m1), GFPT1 (Hs00899865_m1), (Hs01049570_m1), MM9 (Mm00442991_m1), COL1A1 (Hs00164004_m1; Mm01302043_g1), COL3A1 (Hs00943809_m1; Mm00802331_m1), ACTA2 (Hs00426835_g1; Mm01546133_m1), TGFB1 (Hs00998133_m1; Mm01178820_m1), MMP2 (Mm00439498_m1), TIMP3 (Mm00441826_m1). The qPCR reactions were cycled with a pre-incubation of 20 seconds at 95°C followed by 40 cycles of 1 second at 95°C and 20 seconds at 60°C. Relative mRNA expression was calculated from the comparative threshold cycle (Ct) values relative to GAPDH (ΔCt).

#### Bleomycin-induced pulmonary fibrosis

18-month-old (aged) male C57BL/6J mice received a single dose of bleomycin (1.25 U/kg body weight) (UAB ARP Pharmacy) or normal saline via oropharyngeal instillation. Mice were anesthetized with a mixture of 3% isoflurane (VetOne, UAB ARP Pharmacy) and 3% oxygen prior to the procedure. After the instillation, the anesthetized mice were kept on a warm bed for recovery. The experimental animals were monitored daily for adverse clinical signs, including body weight, appearance, hydration status, and any behavioral changes. Mice with concerning weight loss were supplemented with a NutraGel nutrition pack (UAB ARP Pharmacy).

### Oropharyngeal instillation of siOGT in aged mice

Following a single dose of normal saline or bleomycin administration between day 19 and 21, mice were given 8 doses of sham RNA (siCTRL) or siOGT (Horizon Discovery) diluted in 1X PBS every other day via oropharyngeal instillation to randomized groups until euthanized at day 40. Mice were anesthetized for each siRNA treatment as described above. Post treatment care was carried out as described above.

### siRNA constructs

#### siCTRL

Sense: 5’ U.A.A.G.G.C.U.A.U.G.A.A.G.A.G.A.U.A.C.U.U 3’

Antisense: 5’ 5’-P.G.U.A.U.C.U.C.U.U.C.A.U.A.G.C.C.U.U.A.U.U 3’

#### siOGT

Sense: 5’ G.G.C.A.U.G.U.U.A.U.U.U.G.A.A.A.G.C.A.U.U 3’

Antisense: 5’ 5’-P.U.G.C.U.U.U.C.A.A.A.U.A.A.C.A.U.G.C.C.U.U 3’

### Lung homogenization

Lungs were separated by lobes into RINO tubes (cat no: NAVYR1-RNA; Next Advance) and filled with ice cold milliQ water. Lungs were then homogenized using a bullet blender (Bullet Blender 24; Next Advance) at max speed for 1 minute at 4°C followed by placing samples on ice for a 2-minute cool down, then repeat the process for 2-4 more cycles. Samples were centrifuged and inhibitors were added to maintain sample integrity: 1X Halt (1:100, cat no: 78442, Thermo Fisher Scientific), 1X Z-PUGNAc (1:1000, cat no: 3384, Tocris), 1X Thiamet G (1:1000, cat no: 13237; Cayman Chemical) and 1X Benzonase (1:1000, E1014; Millapore Sigma). Homogenized lungs were split for protein, RNA, and hydroxyproline assays.

### Immunohistochemistry and Masson’s trichrome staining

Histological procedures were performed as previously described (43). Briefly, mice were euthanized and lungs were perfused with 5 mL of PBS. Lungs were then inflated and fixed in 10% neutral-buffered formalin (cat no: 57225, Thermo Fisher Scientific) for 24 hours and then switched to 70% ethanol for dehydration and storage. The UAB Histology core processed, sectioned, and stained the lung tissue sections. Lung tissue sections were stained for Masson’s trichrome to detect collagen and O-GlcNAc (RL2; cat no: NB300-524; Novus Biologicals). Images were taken at 4x and 20x magnification on a Nikon ECLIPSE Ts2 Inverted microscope with an image analysis software (NIS-Elements D).

### Drosophila melanogaster conditional knockdown/overexpression of OGT

We crossed Gal4 virgin females to RNAi/GFP/w1118/Sxc-RNAi/UAS-Sxc males to obtain F1 adult flies for experiments. F1 adult flies were collected upon exclusion and then separated into male and female flies in groups of 25-30 on day 3. Flies were transferred onto fresh media every 3 days. All experiments were performed on 3-week-old female flies. Gal4 drivers used include *Act88F-Gal4* (40) which drives largely in indirect flight muscle (IFM) from 24h after puparium formation (APF), *r4-Gal4*, which drives expression in the fat body from late embryo through adult stages (44), and *Ubi-Gal4*, which is a ubiquitous driver (45).

### Immunofluorescence

#### Human lung fibroblasts

An average of 1x10^4^ cells of IMR-90s were seeded on μ-Slide 8 Well^high^ coverslip (cat no: 80806; ibidi) into each well and allowed to attach to the slide overnight in a CO2 (5%) incubator at 37°C. Cells were then serum starved the following day for 12-16 hours. Media was changed back to normal media with 10% FBS for inhibitor treatment with OSMI-4 (10 μM) or TMG (1 μM) for 2 hours followed by TGF-β1 (5 ng/mL) stimulation for 48 hours. Cell culture media was then discarded and cells were fixed with 4% paraformaldehyde (cat no: 15710; Electron Microscopy Sciences) in 1X PBS for 15 minutes at room temperature followed by three 1X PBS washes. Fixed cells were then permeabilized with 0.1% Triton X-100 (cat no: T8787-100ML; Millapore Sigma) in 1X PBS for 20 minutes at room temperature and then washed three times with 1X PBS. Fixed cells were blocked in 1% BSA in 1X PBS at room temperature followed by incubation with primary antibodies: α-SMA-Cy3 (1:100; cat no: C6198; Millapore Sigma) and Collagen I [EPR7785] (1:200; cat no: ab138492; abcam) diluted in 0.1% BSA at 4°C overnight. The primary antibody was discarded the following day and fixed cells were washed with wash buffer [1X PBST (0.05% Tween 20)] three times. Fixed cells were incubated with Alexa Fluor 488 goat anti-rabbit IgG antibody (1:2000; cat no: A-11034; Invitrogen) diluted in 0.1% BSA for 1 hour at room temperature in the dark. Cells were then washed with wash buffer three times and finally with 1X PBS. Chamber was removed using the removal tool and cells were mounted with mounting medium with DAPI (cat no: 50011; ibidi) and cured overnight in the dark at room temperature. Cells were imaged at 60x magnification using the Nikon A1R Confocal Microscope from the UAB High Resolution Imaging Facility.

#### Drosophila melanogaster

To dissect adult fly abdomens, the abdomen was cut open on its ventral side and the gut, gonads, and other inner tissues were removed using forceps. Abdomens were then fixed in 4% PFA for 25 min and washed three times in 1X PBS for 15 min. For pericardin staining, fixed adult abdomens were stained with anti-Pericardin antibody (5ug/ml, 1:10; cat no: EC11; Developmental Biology Hybridoma Bank, University of Iowa) overnight. Samples were then mounted with Diamond Antifade Mountant with DAPI (cat no: P36962; Thermo Fisher Scientific). Confocal images were taken from a Nikon A1R HD microscope (UAB) at 10x for pericardin quantification and representative images. Quantification of pericardin expression from the confocal images from were done using FIJI image analysis software (46). Six squared quadrants of equal area were taken to measure fluorescence intensity from abdominal fat bodies. Squares were split 3:3 on either side of the heart. The average of all six quadrants is reported per specimen.

### Hydroxyproline assay

Total collagen was measured using a hydroxyproline assay kit (cat no: QZBTOTCOL1; QuickZyme Biosciences), and the experiment was performed according to the manufacturer’s instructions. Briefly, mouse lung homogenates were weighed and transferred into screw capped tubes and an equal volume of 12M HCl (cat no: 258148-500ML-GL; Millipore Sigma) was added (final concentration is 6M HCl). Samples were incubated for 20 hours at 95°C.The following day, samples were cooled down to room temperature and centrifuged for 10 minutes at 13,000 x g and supernatant was transferred to a new tube. Hydrolyzed samples were diluted with water down to a final concentration of 4M HCl. Samples were loaded on a 96-well plate with assay buffer and incubated at room temperature for 20 minutes on a plate shaker. Detection reagent was added to each well and the plate was briefly mixed and placed in a 60°C oven for 60 minutes. Plate was cooled on ice for 5 minutes and hydroxyproline concentration was measured at 570 nm using a BioTek plate reader (Agilent, United States).

### Bacteria cloning and protein expression

#### Molecular cloning

Restriction digestion was performed according to manufacturer’s instructions. Briefly, 1 μg of DNA of pET-Duet-hOGT (47) or pCMV6-Entry-Smad3 (cat no: RC208749; Origene) was incubated with AsiSI (10 units; cat no: R0630S; New England BioLabs) and PspXI (5 units; cat no: R0656S; New England BioLabs) for 1 hour at 37°C. Ligation of Smad3 with the pET-Duet-hOGT plasmid was performed according to manufacturer’s instructions. Briefly, T4 DNA ligase (cat no: M0202S; New England BioLabs) was incubated with vector and insert DNA overnight at 16°C. Chemically competent *E. coli* DH5α, NEB Turbo, or BL21 (DE3) was transformed with 1 ng of DNA of pET-Duet-hOGT-Smad3 plasmid according to the manufacturer’s instructions. Engineered cells were then plated onto LB-agar (cat no: IB49101, IBI Scientific) plates with ampicillin antibiotic (100 mg/mL) and allowed to grow in a 37°C incubator overnight. The following day, colonies were selected for protein expression.

#### Protein expression

Transformed BL21 (DE3) *E. coli* cells containing pET-Duet-hOGT-hSmad3 plasmid were grown in a 5 mL LB+ampicillin starter culture at 37°C at 250 rpm overnight in a shaker. Starter culture was used to inoculate a 1 L LB media culture at 37°C at 250 rpm until OD600 ~0.8. To induce protein expression, 0.2 mM of IPTG was added to the culture media for overnight shaking at 250 rpm at room temperature. The inoculated culture was centrifuged at 4,700 rpm for 20 minutes at 4°C to pellet the cells. Cells were lysed with B-PER^®^ Bacterial Protein Extraction (cat no: 78248; Thermo Fisher Scientific), lysozyme (0.2 mg/mL; cat no: 89833; Thermo Fisher Scientific), 1 mM bacterial protease inhibitor cocktail (cat no: P50800-1; RPI), and Benzonase (1 μL/mL) for 30 minutes on ice. Samples were then sonicated on ice with a 10 seconds on, 20 seconds off cycling at 60% amplitude for 12 minutes. Following sonication, samples were ultracentrifuged (Optima XPN-80) at 32,000 rpm for 45 minutes. Isolated proteins in the supernatant was stored at 4°C for short-term use.

### Protein purification

Recombinant proteins from *E. coli* cells were size filtered through Amicon^®^ Ultra-4 Centrifugal Filter unit (cat no: UFC8050; Millipore Sigma). Additionally, this step serves as a buffer exchange to store proteins in 20 mM Tris HCl pH 8, 150 mM NaCl, 1 mM dithiothreitol (DTT). Following size exclusion purification, recombinant proteins were subjected to S-protein Agarose (cat no: 697043; Millipore Sigma) affinity purification to capture recombinant human (rh) Smad3. S-tagged protein purification was performed according to the manufacturer’s instructions using Disposable Plastic Columns (cat no: 29922; Thermo Fisher Scientific). Briefly, S-protein Agarose was added to recombinant proteins and allowed to bind overnight at 4°C. rhSmad3 was eluted using 3 M MgCl_2_ and followed by buffer exchange to store purified rhSmad3.

### Click-iT O-GlcNAc enzymatic labeling, detection, and enrichment

O-GlcNAc enzymatic labeling and detection or azide modified protein capturing was performed as described in the manufacturer’s protocol (cat no: C33368, C33372, and C10416; Thermo Fisher Scientific). Briefly, 200 ug of proteins isolated from human lung fibroblasts were precipitated using the chloroform/methanol method. Proteins were then enzymatically labeled with or without the Gal-T1 (Y289L) overnight at 4°C. Following overnight labeling, the samples were precipitated again using the chloroform/methanol method and azide-labeled proteins underwent Click-iT biotin labeling for western blot applications or alkyne agarose bead enrichment for mass spectrometry analysis.

### Western blot analysis

#### Human lung fibroblasts

As previously described (42, 43), cell lysates were prepared in 1X RIPA buffer (cat no: 9806; Cell Signaling) with 1X Halt protease and phosphatase inhibitor, 1X Z-PUGNAc, 1X Thiamet G, and 1X Benzonase. Protein concentration was measured by Bradford protein assay as previously described. Proteins (30 μg) were separated by SDS-PAGE using a 4-20% precast Ready Gel (Bio-Rad) and transferred to a 0.22 μm or 0.45 μm nitrocellulose membrane. Membrane was rinsed twice with 1x Tris-buffered saline with Tween 20 (cat no: BP337-500; Fisher Scientific) (TBST) then blocked with 5% milk (unless otherwise stated) and incubated with the primary antibodies overnight at 4°C. Primary antibodies used in this study: Pan Collagen (1:800; cat no: PA5-104252, Thermo Fisher Scientific), Col1α1 [EPR22894-89] (1:1000; cat no: ab260043; abcam), Col3α1 (1:1000; cat no: A3795; Abclonal), Pericardin (1:1000; cat no: EC11; Developmental Biology Hybridoma Bank), OGT (1:1000; cat no: GTX109939, Genetex), OGA (1:1000; cat no: A304-345A; Bethyl Lab), Smad3 (1:1000; cat no: 9523S; Cell Signaling), S-tag (1:1000; cat no: 12774S; Cell Signaling), β-actin-HRP (1:10,000; cat no: A3854-200UL; Millapore Sigma), α-Tubulin (1:10,000; cat no: 12G10; Developmental Biology Hybridoma Bank). The next day, primary antibodies were discarded and washed 3 times with 1X TBST and incubated with secondary antibodies: GtαRb IgG-HRP (1:5000; cat no: 31466; Thermo Fisher Scientific), GtαMs IgG-HRP (1:5000; cat no: 31430; Thermo Fisher Scientific), or RbαMsIgM (1:4000; cat no: 31456; Thermo Fisher Scientific) for 1 hour at room temperature. The membrane was washed 3 times with 1X TBST and then developed with enhanced chemiluminescence detection solution for imaging.

Blots for p-Smad3 (1:900; cat no: 9520S; Cell Signaling) and α-SMA (1:1000; cat no: MA5-11547; Thermo Fisher Scientific) were blocked with 5% nonfat milk followed by primary and secondary antibody dilution in 5% BSA.

Blots for O-GlcNAc (1:800; clone CTD110.6; cat no: 838004; Biolegend) were blocked in 3% nonfat milk followed by primary and secondary antibody dilution in 3% BSA.

Blots for Streptavidin-HRP (1:10,000; cat no: SA10001; Thermo Fisher Scientific) were blocked and incubated in primary antibody diluted in 1% nonfat milk.

All blots were imaged using the GE Imaging System (GE Healthcare, USA) and densitometric analyses was performed using FIJI software. All immunoblots were repeated for at least three independent trials with comparable results.

#### Mouse lung homogenates

Briefly, proteins were isolated from mouse lung homogenates using 1X RIPA buffer with Halt protease/phosphatase inhibitor (1:100), Z-PUGNAC (1:1000), Thiamet G (1:1000), and Benzonase (1:1000). Isolated proteins were quantified using the Bradford assay and subjected to SDS-PAGE and western blot as mentioned above.

#### Drosophila melanogaster

Five thoraces were added to 75 µl of lysis buffer (62mM Tris pH7.5, 0.1% SDS with 1X Halt protease and phosphatase inhibitors). The samples were boiled for 5 min at 95°C and centrifuged to collect the supernatant. Laemmli sample loading buffer (cat no: 1610747; Bio-Rad) was added, and the samples were subjected to SDS-PAGE followed by western blot.

#### Immunoprecipitation

Proteins were pre-cleared with protein L agarose (cat no: 20510; Thermo Fisher Scientific) for 1 hour tumbling end over end at 4°C. Following preclearing, proteins were centrifuged briefly and supernatant was transferred into a new tube for incubation with O-GlcNAc (CTD110.6; 1:250) antibody overnight at 4°C. The next day, protein L agarose is added to sample and mixed for 2 hours tumbling end over end at 4°C. Proteins were then centrifuged at 10,000rpm for 1 minute and the supernatant was discarded. The pellet was washed with 1 mL (75 mM Tris pH 7.4, 150 mM NaCl, 1% Triton X-100) four times, vortexing, centrifuging, and aspirating supernatant in between each wash. The pellet was then washed with 1 mL (75 mM Trish pH 6.3) twice following the same steps as before. Samples were then boiled in Laemmli buffer with 2-mercaptoethanol (cat no: m3148; Millapore Sigma) for 10 minutes and stored until gel electrophoresis.

#### Mass spectrometry

For alkyne agarose bead enrichment, once the proteins were bound to the beads, they were reduced and alkylated. This was followed by stringent washing with SDS buffer, 8M urea in Tris buffer and 20% acetonitrile to remove non-specifically bound proteins. The proteins were digested off the beads with an overnight incubation with trypsin. The peptides were subsequently desalted using an ultramicrospin C-18 column (Nest Group, Ipswich, MA) prior to being submitted for mass spectrometry analysis.

A Q‐Exactive HF-X mass spectrometer (Thermo Scientific) was used for mass spectrometry analysis, acquiring datasets for liquid chromatography separation of a 0.1 μg/μL peptide solution loaded on the analytical separation column. The nanoelectrospray voltage was set at 2.2kV, and the ion transfer tube temperature was set to 300°C. Dataset was acquired from 300 to 1800 m/z for 120 minutes, with the data acquisition initiated after a 20.6-minute delay from sample loading onto column (completion of sample trapping and start of elution gradient). A resolution for the 60K (AGC 3e6) was used for the MS1 analysis, followed by a top 12 FT‐HCD‐MS/MS spectra acquired in data‐dependent mode using an isolation window of 0.7 m/z at a resolution of 45 k (AGC target 1e5). A normalized collision energy of 30 was used for HCD fragmentation, analyzing only charge states from 2 to 6, with the exclusion window set to 45 seconds.

A Thermo Dionex Ultimate configured with 2 pumps for sample trapping and reverse-flow elution of the sample onto the analytical column respectively was used for liquid chromatography separation of the peptide sample solution. A 4-cm x 100μm id capillary packed with 5-μm Jupiter C2 media was used as the trap column. A 30-cm × 75 μm i.d. integrated emitter capillary packed with a 1.7 μm particle size C18 media (Waters Acquity BEH particles, Waters) was used for reverse phase peptides elution. Elution was done at a flow rate of 200 nL/min. Buffer A (0.1% formic acid in H_2_O) and buffer B (0.1% formic acid in acetonitrile) were used as solvents, and the elution gradient used is as follows; (min, %B): 0.0, 1.0; 12.6, 8.0; 107.6, 25.0; 117.6, 35.0; 122.6, 75.0; 125.6, 95.0; 131.6, 95.0; 132.6, 50.0; 134.0, 50.0; 134.6, 95.0; 140.6, 95.0; 142.6, 1.0; 190.0, 1.0.

### Quantification and statistical analysis

Statistical analysis description for each experiment can be found in the figure legends. Western blot images were quantified using FIJI and analyzed relative to β-actin. Each qPCR experiment was performed in duplicates or triplicates and analyzed for relative expression by calculating the 2^-ΔCT^ relative to GAPDH expression. Immunofluorescence images were quantified by measuring signal intensity from select regions from each photo. Statistical analyses were performed in Prism 10 (GraphPad). Error bars represent the mean ± standard error of the mean (SEM). Outliers were determined using the 1.5xIQR rule. A classic student t-test or one-way ANOVA with Tukey’s multiple comparison test was performed to assess significance. Statistical significance was always represented as follows: *p<0.05, **p<0.01, ***p<0.001, ****p<0.0001.

## Results

### Hexosamine biosynthetic pathway genes are upregulated in IPF fibroblasts and myofibroblasts

A previous genomic study reported increased expression of *OGT* in IPF human lung tissues compared to lung tissues from healthy controls (48); however, no other characterization of OGT or other proteins involved in the HBP were investigated. Moreover, the specific cell types demonstrating increased *OGT* expression were not identified. To determine the relevance of the HBP and O-GlcNAc in IPF, we analyzed a previously published dataset containing human lung cell scRNA-seq (single-cell RNA-sequencing) data (GSE147066) from IPF (n=32) and non-IPF donors (n=29) for genes in the HBP (**Figure 1A**) (41). Cell populations were stratified based on expression of *CTHRC1* (collagen triple helix repeat containing 1) by the original authors and four subpopulations were identified (**Figure 1B**). Based on the spatial mapping, the fibroblast and myofibroblast cell populations had higher expression of collagens, *COL1A1 and COL3A1*, compared to the pericyte and smooth muscle cell populations (**Supplementary Figure 1**). In addition, spatial cluster maps of *GFPT1* (GFAT; the rate-limiting step in the HBP) (49), *OGT* and *MGEA5* (gene encoding OGA) showed more expression in the selected IPF fibroblast and myofibroblast cell populations that had increased collagen expression (*COL1A1 and COL3A1)* and other fibrotic markers (*ACTA2* and *TGFB1*) compared to the non-IPF controls (**Figure 1C**). Consistent with the public scRNA-seq data, our mRNA expressions of *OGT*, *MGEA5*, *GFPT1*, *COL1A1*, *COL3A1*, *ACTA2*, and *TGFB1* from IPF human lung fibroblasts (HLFs) were all significantly increased compared to non-IPF donors (**Figures 1D-J**). Collectively, these data indicate that increased expression of HBP markers (*OGT*, *MGEA5*, and *GFAT1*) is observed in IPF fibroblast and myofibroblast cell populations with augmented collagen expression.

### Increased global O-GlcNAc levels in IPF lungs and mouse lungs of bleomycin-induced fibrosis

**Figure 2:**
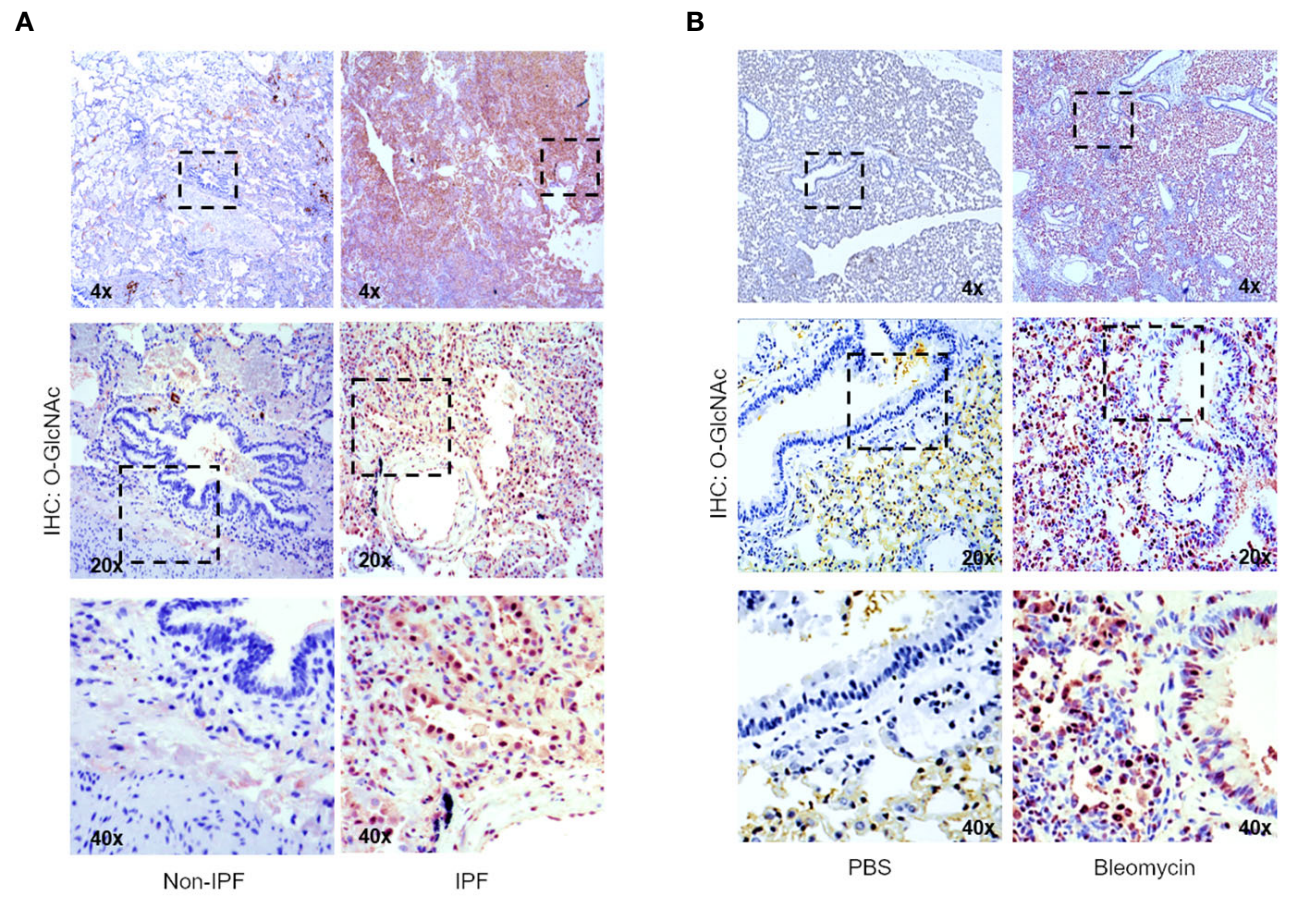
Increased global O-GlcNAc levels in IPF lungs and mouse lungs of bleomycin-induced fibrosis. **(A)** Representative IHC of lung tissue sections from non-IPF and IPF donors stained for O-GlcNAc (RL2); 4x, 20x, 40x magnification. **(B)** Representative IHC of mouse lung tissue sections stained for O-GlcNAc (RL2) 3 weeks post saline or bleomycin treatment; 4x, 20x, 40x magnification.

To determine the levels of the O-GlcNAc modification in IPF lung tissue, non-IPF and IPF lung tissues were subjected to IHC staining with antisera against O-GlcNAc (**Figure 2A**). Increased HRP staining of O-GlcNAc was observed in fibrotic regions of the parenchyma in IPF lung tissue compared to non-IPF controls. Increased O-GlcNAc staining was also observed following bleomycin-induced lung fibrosis in mice compared to saline controls (PBS), a shared feature with our findings in the human tissue (**Figure 2B**). These data indicate that global O-GlcNAc levels are increased in fibrotic regions of lung parenchyma in both IPF and an experimental model of pulmonary fibrosis.

### OGT regulates collagen accumulation *in vivo*

**Figure 3:**
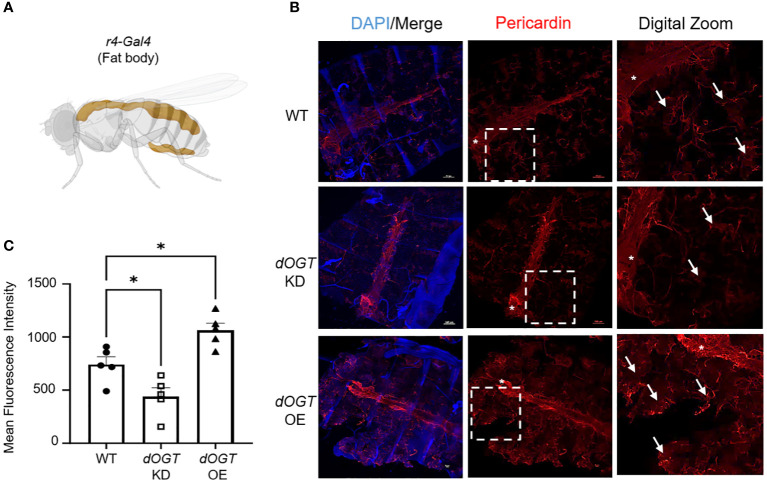
OGT regulates collagen accumulation *in vivo*. **(A)** Schematic of *r4-Gal4* driver used in *Drosophila melanogaster* experiments for fat body-specific *dOgt* knockdown (KD) or overexpression (OE). Image created with Biorender. **(B)** Representative immunofluorescence staining of pericardin (red) deposition in the fat bodies of *D. melanogaster*; DAPI (blue). **(C)** Mean fluorescence intensity of pericardin deposition from control, *dOgt* KD, and *dOgt* OE, n = 5. Arrowheads indicate fat body regions of high pericardin staining. Asterisk (*) denotes pericardin staining in the heart that was not included in data analysis since the *r4-Gal4* driver is specific to the fat bodies. Data from multiple replicates are presented as mean ± SEM, each dot represents independent biological replicate. Statistical analyses were done using the Student’s t-test. *p<0.05.

To investigate the effect of gain- and loss-of-function of OGT on collagen accumulation, we performed overexpression (OE)/knockdown (KD) of the *Drosophila* homolog of *Ogt*, *Sxc* (referred to as *dOgt*), in *Drosophila melanogaster* and examined the consequences on a type IV collagen-like protein, pericardin, expression. Since the flies do not have lungs, we examined the expression of pericardin in the fat bodies of *Drosophila*, which are liver- and adipose-like tissue in the abdomen, to objectively determine the role of OGT on collagen accumulation *in vivo* (50, 51). We conducted whole-body (*Ubi-Gal4*), indirect flight muscle (*Act88F-Gal4*), and fat body-specific (*r4-Gal4*) KD or OE of *dOgt* (**Supplementary Figure 2A**; **Figure 3A**). Global O-GlcNAc levels and dOGT protein expression from whole-body and thorax upon respective manipulations showed that *dOgt* KD or OE can reduce or increase O-GlcNAc and dOGT expression, respectively (**Supplementary Figures 2B-G**). Furthermore, confocal microscopy imaging demonstrated that pericardin deposition in the fat bodies was significantly decreased following *dOgt* KD, but significantly increased upon *dOgt* OE compared to the control flies (**Figures 3B, C**). Altogether, these data suggest that OGT regulates collagen-like protein accumulation *in vivo*, and may be a critical component of the ECM remodeling and fibrotic process in higher order species.

### OGT knockdown reverses collagen deposition in a mouse model of non-resolving fibrosis

**Figure 4:**
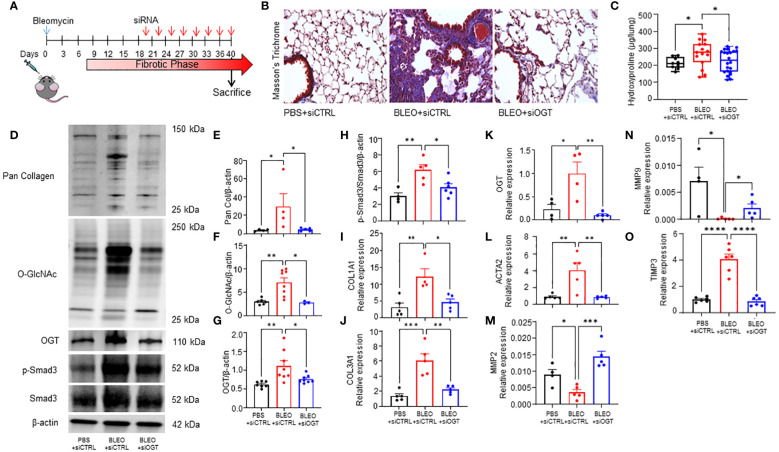
OGT knockdown reverses collagen deposition in a mouse model of non-resolving fibrosis. **(A)** Schematic illustrating the timelines for administration of bleomycin and siRNA and determination of experimental end points in the non-resolving model of pulmonary fibrosis. **(B)** Representative Masson’s trichrome staining for collagen deposition (blue), cytoplasm/erythrocytes/muscles (red), merge (purple); 20x magnification. **(C)** Effects of bleomycin and OGT knockdown following bleomycin treatment in mice, as reflected by changes in total hydroxyproline content, n = 9-22, **(D)** representative western blot images of pan collagen, O-GlcNAc, OGT, p-Smad3, Smad3, and β-actin, **(E-H)** densitometric graphs of the ratio of pan collagen, O-GlcNAc, OGT, p-Smad3, and Smad3 to β-actin, n = 4-8, (**I–O**) and the mRNA expression of OGT and fibrotic markers (*COL1A1, COL3A1, OGT, ACTA2, MMP2, MMP9, TIMP3*), n = 4-6. Values from hydroxyproline, western blot, and qPCR represent results from three pooled independent experiments. Data from multiple replicates are presented as mean ± SEM, each dot represents independent biological replicate. Outliers determined by the 1.5xIQR rule were excluded, and statistical analyses were done using the Student’s t-test and one-way ANOVA with Tukey’s multiple comparisons test. *p<0.05, **p<0.01, ***p<0.001, ****p<0.0001.

Since we observed increased O-GlcNAc in human and mouse pulmonary fibrosis and regulation of collagen expression by OGT in *D. melanogaster*, we wanted to determine the effects of reducing O-GlcNAc on fibrosis resolution. Aged mice (18 months), an established model for non-resolving pulmonary fibrosis (52), were administered 8 doses of OGT siRNA (siOGT), via oropharyngeal instillation, to knockdown OGT following peak fibrosis or 3 weeks post-bleomycin treatment (**Figure 4A**) (53). Collagen deposition/fibrosis 6-weeks post bleomycin (BLEO+siCTRL) was increased compared to controls (siCTRL). Strikingly, oropharyngeal-delivered KD of OGT post-peak fibrosis resulted in significant reduction in collagen deposition as determined by Masson’s trichrome staining and quantification of lung collagen content by measurement of total hydroxyproline from acid hydrolyzed lung homogenate (**Figures 4B, C**). Immunoblots for pan collagen, O-GlcNAc, OGT, phosphorylated Smad3 (p-Smad3), and total Smad3 validated our findings and fibrotic markers were elevated in the BLEO+siCTRL group compared to the BLEO+siOGT group, which demonstrated similar levels to baseline (siCTRL) (**Figures 4D-H**). Transcript analyses of *COL1A1, COL3A1, OGT*, and *ACTA2* showed a similar upregulation trend with BLEO+siCTRL treatment and downregulation of each gene after siOGT treatment (**Figures 4I-L**). Transcript analyses of the collagenases, *MMP2* and *MMP9*, exhibited a contrasting trend to *ACTA2* and collagen suggesting a suppression of *MMP2,-9* expression during fibrosis that is upregulated following OGT knockdown (**Figures 4M, N**). Furthermore, *TIMP3* (tissue inhibitor of metalloprotease 3) increased during fibrosis and was reduced following OGT KD (**Figure 4O**). These data indicate that OGT regulates collagen expression and fibrosis resolution in a pre-clinical model of pulmonary fibrosis.

### Pharmacological manipulation of O-GlcNAc regulates collagen level in a TGF-β1-induced FMT model

**Figure 5:**
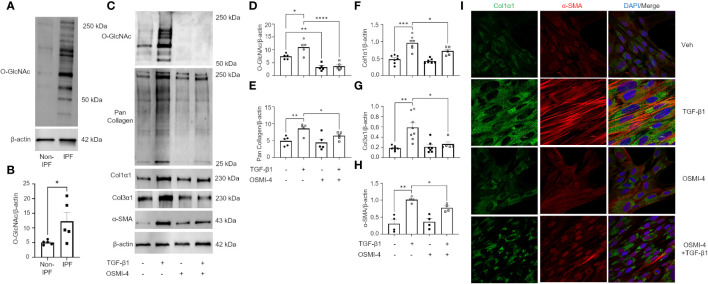
Pharmacological inhibition of OGT attenuates TGF-β1 induction of collagen expression and α-SMA (markers of FMT). **(A)** Representative western blot images of O-GlcNAc and β-actin from IPF and non-IPF primary HLFs. **(B)** Densitometric graphs of the ratio of O-GlcNAc to β-actin, n = 5. **(C)** Representative western blot images of O-GlcNAc, pan collagen, Col1α1, α-SMA, and β-actin expression from HLFs with or without OSMI-4 inhibition (10 μM) followed by TGF-β1 (5 ng/mL) stimulation for 48 hours. **(D–H)** Densitometric graphs of the ratio of O-GlcNAc, pan collagen, Col1α1, Col3α1, α-SMA, to β-actin, n = 5-7. **(I)** Representative immunofluorescence staining of Col1α1 (green) and α-SMA (red) expression; DAPI (blue). Data from multiple replicates are presented as mean ± SEM, each dot represents independent biological replicate. Outliers determined by the 1.5xIQR rule were excluded, and statistical analyses were done using the Student’s t-test and one-way ANOVA with Tukey’s multiple comparisons test. *p<0.05, **p<0.01, ***p<0.001, ****p<0.0001.

**Figure 6:**
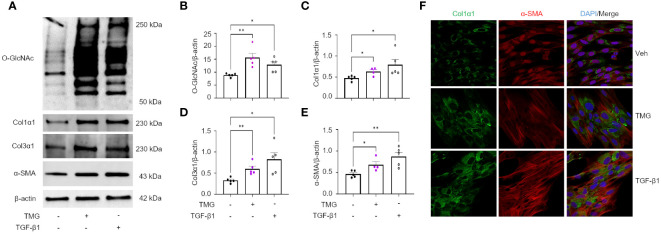
OGA inhibition drives the expression of key FMT markers. **(A)** Representative western blot images of O-GlcNAc, Col1α1, Col3α1, α-SMA, and β-actin expression from HLFs with TMG (1 μM) or TGF-β1 (5 ng/mL) stimulation for 48 hours. **(B–E)** Densitometric graphs of the ratio of O-GlcNAc, Col1α1, Col3α1, α-SMA, to β-actin, n = 4-5. **(F)** Representative immunofluorescence images of Col1α1 (green) and α-SMA (red) expression; DAPI (blue). Data from multiple replicates are presented as mean ± SEM, each dot represents independent biological replicate. Outliers determined by the 1.5xIQR rule were excluded, and statistical analyses were done using the Student’s t-test. *p<0.05, **p<0.01.

The TGF-β pathway is a master regulator of fibrosis and the TGF-β1-induced fibroblast-to-myofibroblast transition (FMT) has been documented in fibrosis and used as an *in vitro* method to mechanistically study fibrogenesis (54, 55). To determine the role of O-GlcNAc in ECM remodeling and FMT, HLFs were treated with TGF-β1 in the presence and absence of OGT inhibitor (OSMI-4). TGF-β1 administration increased global O-GlcNAc levels in HLFs similar to the levels observed in primary IPF lung fibroblasts (**Figures 5A-D**). OGT inhibitors (OSMI-4, OSMI-1, Ac_4_5S-GlcNAc) abrogated the effects of TGF-β1 induction on O-GlcNAc levels and upregulated OGT expression while concurrently downregulating OGA expression as a feedback response to the inhibition (**Figures 5C, D**; **Supplementary Figures 3A-E, H, I**). Blocking O-GlcNAc transfer attenuated TGF-β1 induction of FMT as determined by the expression of pan collagen (**Figures 5C, E**), Col1α1 (**Figures 5C, F**; **Supplementary Figure 3D, F, H, J**), Col3α1 (**Figures 5C, G**), and α-SMA (**Figures 5C, H**; **Supplementary Figure 3D, G, H, K**), all of which were significantly reduced. Validation of these observations were shown by immunofluorescence staining of Col1α1 and α-SMA (**Figure 5I**). Conversely, the opposite result was observed following pharmacological inhibition of OGA [Thiamet G, (TMG); increases O-GlcNAc] where O-GlcNAc, Col1α1, Col3α1, and α-SMA protein expression were significantly increased compared to vehicle control, and congruous with TGF-β1 induction (**Figures 6A-E**). Furthermore, immunofluorescence images validate our prior observations (**Figure 6F**). Collectively, these data demonstrate that the OGT/O-GlcNAc axis regulates collagen (and α-SMA) expression *in vitro* and may be a key mediator in the FMT, a process central to pulmonary fibrosis.

### Click-iT™ O-GlcNAc capture and proteomic analysis of HLFs identified multiple O-GlcNAc modified proteins including Smad3

**Figure 7:**
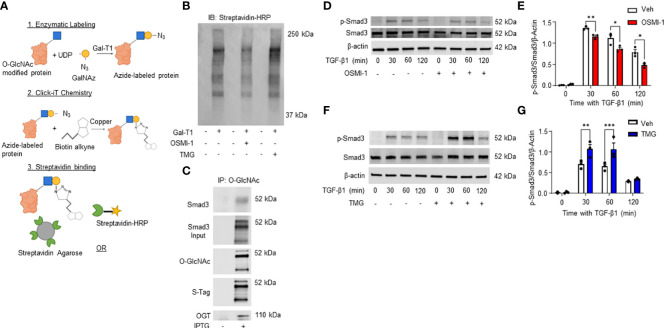
Click-iT^(R)^ O-GlcNAc capture and proteomic analysis of HLFs identifies multiple O-GlcNAc modified proteins including Smad3. **(A)** Schematic illustrating Click-iT labeling of O-GlcNAc modified proteins for western blot or mass spectrometry analysis. Images were created with Biorender. **(B)** Representative streptavidin-HRP blot of O-GlcNAc modified proteins biotinylated through Click-iT^®^ labeling/detection in the presence or absence of OSMI-1 (25 μM), TMG (1 μM), and GalT1 (Y289L). **(C)** Representative western blots images of Smad3 (IP and expression), O-GlcNAc, S-tag, and OGT from an *E. coli* bacterial co-expression system induced with or without IPTG (0.2 mM). **(D, F)** Representative western blot images of p-Smad3, Smad3, and β-actin from HLFs pre-treated with OSMI-1 (25 μM) or TMG (1 μM) followed by time dependent TGF-β1 stimulation (5 ng/mL). **(E,G)** Densitometric graphs of the ratio of p-Smad3 to Smad3 to β-actin. Data from multiple replicates are presented as mean ± SEM, each dot represents independent biological replicate. Statistical analyses were done using the Student’s t-test. *p<0.05, **p<0.01, ***p<0.001. Blue squares = O-GlcNAc sugar. Yellow circles = GalNAz sugar. Green sectors = streptavidin.

Our data demonstrates a role for OGT and the O-GlcNAc modification in FMT and collagen expression/accumulation, and fibrosis resolution. However, specific proteins modified by O-GlcNAc have not been determined. Therefore, we subjected HLFs to Click-iT™ O-GlcNAc Enzymatic Labeling and Detection/Enrichment and mass spectrometry (MS) analysis (**Figures 7A, B**). MS analysis identified over 2,500 O-GlcNAc modified proteins (**Supplementary Table 1**). Furthermore, several proteins enriched from the O-GlcNAc purification were associated with TGF-β signaling including TGF-β1 and α-SMA (**Table 1**). Smad4 and Smad5, which have been documented as O-GlcNAc modified proteins (56, 57), were also identified in the analysis. A novel finding was that Smad3 contained the O-GlcNAc modification. To validate this finding, we inserted a SMAD3 gene into a pET-Duet bacterial plasmid (flanked by an affinity S-tag) containing human OGT. Co-expression of OGT and Smad3 in BL21 DE3 *E. coli* confirmed that Smad3 is modified by OGT by immunoprecipitation (**Figure 7C**). These findings are the first to demonstrate Smad3 as an O-GlcNAc modified protein, which may have implications in Smad activation and downstream targets associated with FMT and fibrosis.

**Table 1:** O-GlcNAc modified proteins identified by mass spectrometry involved in TGF-β signaling and fibrosis.

Protein Name	Gene	Protein ID	Spectral Count(s)	Previously Reported?
Smad3	SMAD3	P84022	1	No
Smad4	SMAD4	Q13485	5	Yes (56)
Smad5	SMAD5	Q99717	2	Yes (57)
α-SMA	ACTA2	P62736	4	No
TIMP1	TIMP1	P01033	4	No
TIMP3	TIMP3	P35625	2	No
OGT	OGT	O15294	3	Yes (58–63)
OGA	MGEA5	O60502	11	Yes (64–66)

### Phosphorylation of Smad3 by TGF-β1 is regulated by the O-GlcNAc modification

TGF-β1 signaling through the Smad3 pathway is well documented in fibrosis (67–71). However, the regulation of Smad3 phosphorylation by O-GlcNAc modification has not been determined. Therefore, we treated HLFs with TGF-β1 in the presence or absence of OGT inhibition. Phosphorylation of Smad3 (p-Smad3; Ser-423/425) was significantly reduced following TGF-β1 administration in primary HLFs when OGT was inhibited (**Figures 7D, E**). Reciprocally, when OGA was blocked to increase O-GlcNAc levels, p-Smad3 levels were enhanced with TGF-β1 stimulation compared to TGF-β1 stimulation alone (**Figures 7F, G**). Taken together, these findings suggest that the O-GlcNAc modification of Smad3 enhances its phosphorylation and may be critical for control of downstream effectors regulated by TGF-β1/Smad3.

## Discussion

**Figure 8:**
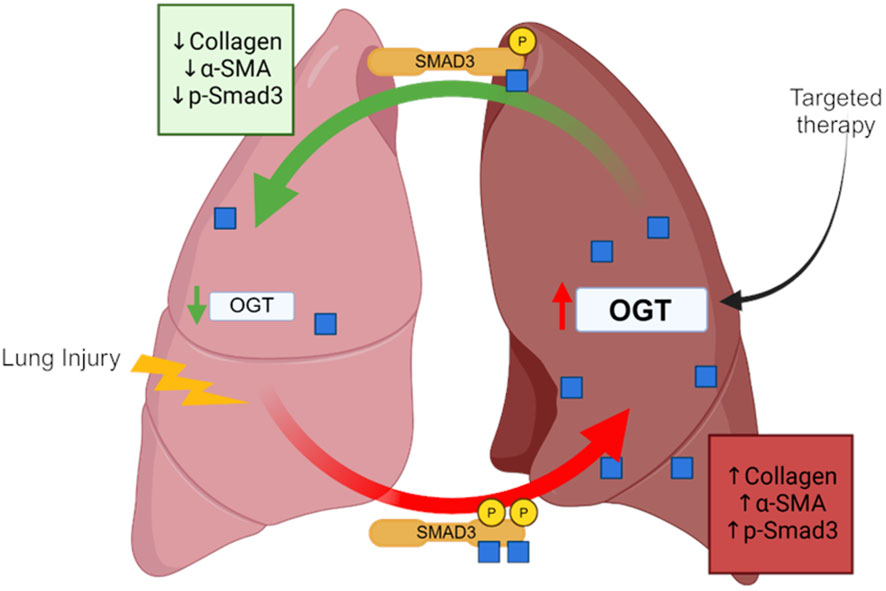
Schematic of the OGT/O-GlcNAc axis regulating collagen expression in pulmonary fibrosis. Image was created with Biorender.

In this study, we show that global O-GlcNAc levels are elevated in IPF lung tissue and modulating O-GlcNAc, via overexpression, siRNA or pharmacological inhibition, was shown to either promote or attenuate collagen expression across multiple species. Furthermore, we are the first to demonstrate that reducing O-GlcNAc, via oropharyngeal knockdown of OGT, resulted in resolution of fibrosis in a non-resolving murine model of pulmonary fibrosis. Upon further investigation, we identified several novel O-GlcNAc modified proteins in human lung fibroblasts that may be involved in fibrosis including Smad3, which directly regulates collagen in fibrosis/resolution. Our data shows that inhibiting O-GlcNAc addition resulted in decreased phosphorylation at Ser-423/425 attenuating the effects of TGF-β1-induced collagen expression/deposition, while increasing O-GlcNAc levels showed the opposite trends (**Figure 8**). These data identify OGT as a potential target in pulmonary fibrosis resolution, as well as other diseases that might have aberrant ECM/collagen accumulation.

Dysregulated protein glycosylation, especially the O-GlcNAc modification, has been documented in chronic lung disease (72–75). We have previously reported that OGT/O-GlcNAc regulates pulmonary arterial smooth muscle cell proliferation and vascular angiogenesis in idiopathic pulmonary arterial hypertension (IPAH) (72). In addition, elevated O-GlcNAc has also been widely reported in lung cancer and in EMT (76–79). These findings have been linked to increased glucose metabolism and metabolic shifts in glycolysis (Warburg effect), which both have been documented during the metabolic reprogramming of fibroblasts to myofibroblasts in IPF (21, 22, 80, 81). In this report, we found that O-GlcNAc levels were higher in IPF lung explants compared to non-IPF lungs (**Figure 2**). In addition, several genes within the HBP had increased expression in IPF cell populations (fibroblast and myofibroblasts) with high collagen expression (**Figure 1**). We speculate that the increase in O-GlcNAc, as well as the HBP genes, is initiated by lung injury and is sustained in these patients. Our models support this concept given that examination of mouse lungs 3 weeks (**Figure 2**) and 6 weeks (**Figure 4**) post-lung injury demonstrated similar findings in sustained O-GlcNAc augmentation. These data suggest that a sustained increased in O-GlcNAc is involved in chronic lung diseases and may be a potential target for treatment of IPF.

O-GlcNAcylation is a dynamic process and aberrant shifts in O-GlcNAc homeostasis (e.g., hyper- vs hypo-O-GlcNAc levels) is a phenomenon that has been described in disease progression (34, 35, 82, 83). While acute changes in O-GlcNAc, similar to phosphorylation, regulate many normal cellular and protein functions, sustained or chronic O-GlcNAc dysregulation may initiate or perpetuate disease processes. This has been widely studied in the cardiovascular system where chronic elevation of O-GlcNAc has been shown to aggrandize cardiovascular complications (36, 84, 85), while acute increases in O-GlcNAc have been shown to be protective against ischemia/reperfusion injury (86–88). Much can be gleaned from recent studies of fibrosis where changes to O-GlcNAc levels have been shown to contribute to disease pathology. For example, complete knockout of OGT (loss of O-GlcNAc) in the liver resulted in fibrosis/cirrhosis, while increased O-GlcNAc was shown in a mouse model of renal fibrosis (34, 35). More interestingly, a report recently published by Yu et al. described that inducible whole-body knockout of OGT in mice resulted in the development of pulmonary fibrosis (89). We demonstrate that increased O-GlcNAc levels are a clinical feature of pulmonary fibrosis (**Figure 2**). Even though these reports highlight the impact and consequence of persistent gain of O-GlcNAc or knock-out of OGT in fibrosis, the approaches did not attempt to correct global levels of O-GlcNAc to homeostasis/basal levels. We were, however, able to reduce the increased OGT protein expression and O-GlcNAc levels to basal levels in a non-resolving fibrosis mouse model using oropharyngeal-delivered siRNA against OGT (**Figure 4D**). The reduction to baseline levels led to partial reversal of fibrosis as determined by hydroxyproline and fibrotic marker expression analysis (**Figures 4B, C**). These data suggest that restoring O-GlcNAc homeostasis is sufficient to halt the accumulation of collagen in fibrosis and reverse the existing deposition.

Collagen regulation is a critical process in the initiation and progression of fibrosis, and targeting collagen production and degradation may offer potential therapeutic strategies for pulmonary fibrosis. In particular, targeting the turnover/degradation of collagen, to promote resolution, has garnered high interest in fibrosis research. At the cellular level, reports have shown that new collagen, produced by myofibroblasts through FMT, exceeds the rate of degradation; therefore, targeting collagen synthesis may be a potential therapeutic strategy for fibrosis (90–92). Here, we report that pharmacological inhibition of OGT attenuates TGF-β1 induced collagen expression and FMT (**Figure 5**), while blocking OGA (increases O-GlcNAc) led to increased collagen (**Figure 6**). This finding was consistent with our *in vivo* data where gain- and loss-of OGT function subsequently caused global changes in accumulation of a collagen-like protein in *D. melanogaster* (**Figure 3**). These findings strongly indicate a role for O-GlcNAc in collagen regulation that is evolutionarily conserved. In line with our findings, several reports have hinted at a potential association between O-GlcNAc and collagen expression. In cardiac fibroblasts, high glucose was used to stimulate the HBP and O-GlcNAc levels and concomitant increases in collagen synthesis was observed (93, 94). In addition, another study showed that increased O-GlcNAc induced chondrogenic differentiation both *in vitro* and *in vivo* (95), suggesting a potential role for O-GlcNAc in the formation of chondrocytes and cartilaginous tissues. On the other hand, cardiomyocyte specific deletion of OGT alone was not sufficient to cause hypertrophy and collagen accumulation in the heart (96). However, infarcted mice with OGT deletion had significantly exacerbated cardiac dysfunction, suggesting that combined effects of injury and an imbalance in O-GlcNAc was needed to elicit exacerbated trauma (96). Our studies provide evidence for the combination of injury and sustained levels of O-GlcNAc in the direct regulation of collagen and fibrosis threefold: 1- in Drosophila melanogaster; 2- in a non-resolving fibrosis mouse model; and 3- in a cell culture model of TGF-β1-induced FMT.

Unanswered questions remain, including the mechanism(s) whereby OGT/O-GlcNAc regulates collagen accumulation and degradation; which proteins involved in fibrosis are modified by O-GlcNAc, and whether OGT is a modifiable target in lung fibrosis. Using the Click-iT^®^ O-GlcNAc enzymatic labeling/detection system, we enriched for O-GlcNAc modified proteins in human lung fibroblasts (**Figure 7**). We identified over 2,500 O-GlcNAc modified proteins including α-SMA, TIMP1, TIMP3, Smad4, Smad5 and Smad3 have been implicated in pulmonary fibrosis (**Figure 7**, **Table 1**). O-GlcNAc has been shown to stabilize the co-Smad protein, Smad4, which is a major regulator of TGF-β signaling (56). To our knowledge, α-SMA, and TIMP1/3 have not been identified as O-GlcNAc modified, but have been documented in fibrosis and fibrosis resolution, consistent with our findings (**Figure 4**), and further underscore a potential benefit of targeting O-GlcNAc in IPF (97, 98). We also report for the first time that Smad3 is a bona fide O-GlcNAc modified protein and is essential for phosphorylation of Smad3 at Ser-423/425. Based on this, we postulate that the O-GlcNAc site must be in proximity with Ser-423/425 because blocking OGT or OGA resulted in reduced or enhanced phosphorylation at Ser-423/425, respectively (**Figures 7**, **4D**). This finding is in line with previous reports suggesting the relationship between O-GlcNAc and phosphate can be allosteric regulation versus site occupancy competition (27, 99). We believe that O-GlcNAc is important for augmenting the phosphorylation of Smad3 and promoting induction of collagen gene expression and fibrosis in IPF (**Figures 4**–**6**). Smad3 specific inhibitors have been shown to abrogate its phosphorylation and downregulate expression of FMT markers (α-SMA and Col1α1) similar to our OGT inhibition data (100, 101). Altogether, our data further confirms a mechanistic role for O-GlcNAc in the regulation of collagen in IPF and advocates for more research to explore OGT/O-GlcNAc as a modifiable target in slowing pulmonary fibrosis and fibrosis resolution.

## Conclusion

In summary, we have uncovered a novel mechanism whereby the OGT/O-GlcNAc axis regulates collagen deposition/resolution both *in vivo* and *in vitro* and demonstrated that collagen regulation by OGT is evolutionarily conserved. Restoring O-GlcNAc to normal levels is sufficient to reverse the deposition and halt accumulation of collagen in fibrosis. We postulate that the regulation of collagen expression is, in part, due to the direct O-GlcNAc modification of Smad3, which enhances its phosphorylation. Further research is warranted to reverse fibrosis and increase survival for patients with IPF by improving delivery methods that target OGT in the lung.

## Limitations of the study

Our study is not without limitations. We used oropharyngeal delivered siRNA to silence OGT in the lung following bleomycin. This method does not directly target a specific cell type in the lung. However, it circumvents issues pertaining to the embryonic lethality of OGT knockout in mammals as well as aging any cell-type specific mouse model and could serve as a future therapeutic way to deliver drugs targeting OGT. Furthermore, our Click-iT^®^ O-GlcNAc enzymatic labeling/detection approach also has limitations. It is possible that the enzyme used in the labeling kit, which is a permissive mutant β-1,4-galactosyltransferase [Gal-T1 (Y289L)] that transfers azido-modified galactose (GalNAz) from UDP-GalNAz to *O*-GlcNAc residues, may produce artifacts. For example, collagen proteins were detected by MS following click-it capture and purification, suggesting that they bear O-GlcNAc. GalT enzymes are known to modify collagens at (hydroxy)proline and (hydroxy)lysine residues and could add the azido (GalNAz) to these residues, thus generating a false positive in our MS dataset. Lastly, our translational results were generated from a single center study and contained a relatively small sample size. However, the limitation of a small sample size was offset by the analysis of a transcriptomic dataset from a well-characterized, previously published larger sample population, as well as human lung fibroblast cell lines that demonstrated consistent results.

## Data availability statement

The Click-iT proteomics data from HLFs represented in the study are deposited in the MassIVE database, accession number MSV000093453. The accession number for the publicly available scRNA-seq can be found in the methods section. All immunofluorescence, immunohistochemistry, and immunoblots reported in this paper will be shared by the corresponding author upon request.

## Ethics statement

The studies involving humans were approved by UAB Institutional Review Board. The studies were conducted in accordance with the local legislation and institutional requirements. Written informed consent for participation was not required from the participants or the participants’ legal guardians/next of kin in accordance with the national legislation and institutional requirements. The animal study was approved by Institutional Animal Care and Use Committee at UAB. The study was conducted in accordance with the local legislation and institutional requirements.

## Author contributions

SV: Conceptualization, Data curation, Formal analysis, Funding acquisition, Investigation, Methodology, Resources, Validation, Writing – original draft, Writing – review & editing. EH: Data curation, Investigation, Writing – review & editing. YG: Data curation, Investigation, Writing – review & editing. BB: Data curation, Investigation, Writing – review & editing. ER: Data curation, Writing – review & editing. ME: Data curation, Writing – review & editing. SB: Data curation, Writing – review & editing. MH: Data curation, Writing – review & editing. EM: Writing – review & editing. LJ: Writing – review & editing. PH: Writing – review & editing. VR: Data curation, Writing – review & editing. RD: Data curation, Writing – review & editing. PC: Data curation, Writing – review & editing. IKA: Data curation, Methodology, Writing – review & editing. HO: Data curation, Methodology, Writing – review & editing. GC: Data curation, Methodology, Writing – review & editing. GM: Conceptualization, Investigation, Writing – review & editing. SK: Conceptualization, Investigation, Writing – review & editing. JB: Conceptualization, Funding acquisition, Investigation, Methodology, Resources, Supervision, Visualization, Writing – original draft, Writing – review & editing.

## Supplementary material

Supplementary Figure 1 Collagen expression is highest in fibroblast and myofibroblast cells. Spatial mapping of pericytes, smooth muscle cells, fibroblasts and myofibroblasts expressing *COL1A1* and *COL3A1* based on scaled expression from 32 IPF donors compared to 29 non-IPF donors. Lighter colors denote lower expression, darker colors denote higher expression, and gray denotes no expression.

Supplementary Figure 2 Overexpression or knockdown of OGT in the whole body or thorax of Drosophila melanogaster. **(A)** Schematic of drivers for tissue-specific *dOGT* knockdown or overexpression used in Drosophila melanogaster experiments. Images were created with Biorender. **(B)** Representative western blot images of O-GlcNAc, *dOGT*, and α-Tubulin expression from Ubi-Gal4-driven *dOGT* KD or OE flies. Black line indicates spliced-together lanes, however, the lanes were run on the same gel, but were noncontiguous. **(C–D)** Densitometric graphs of the ratio of O-GlcNAc and *dOGT* to α-Tubulin from Ubi-Gal4-driven *dOGT* KD or OE flies, n = 2-5. Whole-body *dOGT* KD hindered growth and viability of fruit flies; therefore, biological replicates were limited. **(E)** Representative western blot images of O-GlcNAc, *dOGT* and α-Tubulin expression from Act88f-Gal4-driven *dOGT* KD or OE flies. **(F–G)** Densitometric graphs of the ratio of O-GlcNAc and *dOGT* to α-Tubulin from Act88f-Gal4-driven *dOGT* KD or OE flies, n = 4. Data from multiple replicates are presented as mean ± SEM, each dot represents independent biological replicate. Statistical analyses were done using the Student’s t test. *p<0.05, **p<0.01, ****p<0.0001.

Supplementary Figure 3 OSMI-1 and 5S-GlcNAc, two different OGT inhibitors, attenuate TGF-β1 induction of FMT markers, similar to OSMI-4. **(A)** Representative western blot images of OGT, OGA, and β-actin expression from HLFs with or without OSMI-4 inhibition (10 µM) followed by TGF-β1 (5 ng/mL) stimulation for 48 hours. **(B, C)** Densitometric graphs of the ratio of OGT and OGA to β-actin, n = 4-5. **(D)** Representative western blot images of O-GlcNAc, Col1α1, α-SMA, and β-actin expression from HLFs with or without OSMI-1 inhibition (25 µM) followed by TGF-β1 (5 ng/mL) stimulation for 48 hours. **(E–G)** Densitometric graphs of the ratio of O-GlcNAc, Col1α1, α-SMA, to β-actin, n = 5-6. **(H)** Representative western blot images of O-GlcNAc, Col1α1, α-SMA, and β-actin expression from HLFs with or without 5S-GlcNAc inhibition (50 µM) followed by TGF-β1 (5 ng/mL) stimulation for 48 hours. **(I–K)** Densitometric graphs of the ratio of O-GlcNAc, Col1α1, α-SMA, to β-actin, n = 5-6. Black line indicates spliced-together lanes, however, the lanes were run on the same gel, but were noncontiguous. Data from multiple replicates are presented as mean ± SEM, each dot represents independent biological replicate. Outliers determined by the 1.5 IQR rule were excluded, and statistical analyses were done using the Student’s t-test. *p<0.05, **p<0.01, ***p<0.001, ****p<0.0001.
