# Supplementary figures and images for: O-GlcNAc transferase regulates collagen deposition and fibrosis resolution in idiopathic pulmonary fibrosis
Source: Front Immunol. 2024 Apr 11;15:1387197. doi: 10.3389/fimmu.2024.1387197 (PMC11043510; doi:10.3389/fimmu.2024.1387197)

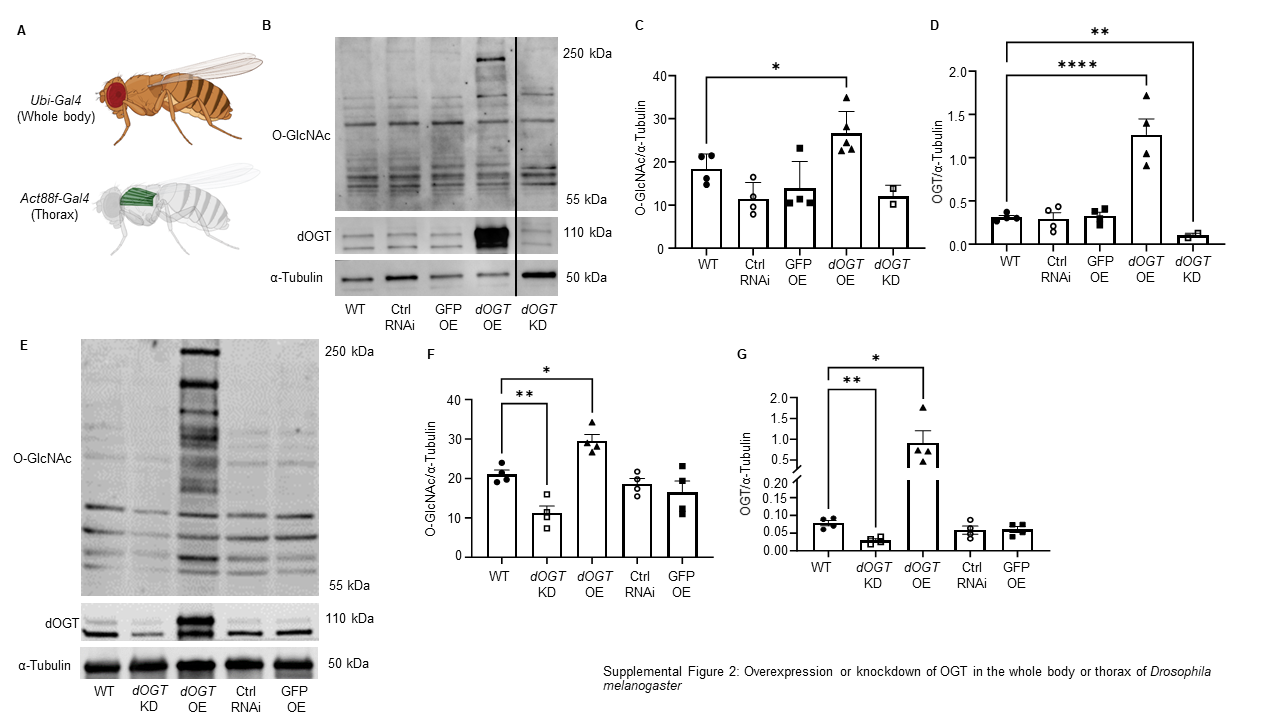

Supplement: Supplementary Figure 1 — Collagen expression is highest in fibroblast and myofibroblast cells. Spatial mapping of pericytes, smooth muscle cells, fibroblasts and myofibroblasts expressing COL1A1 and COL3A1 based on scaled expression from 32 IPF donors compared to 29 non-IPF donors. Lighter colors denote lower expression, darker colors denote higher expression, and gray denotes no expression. [file Image_1.tif]

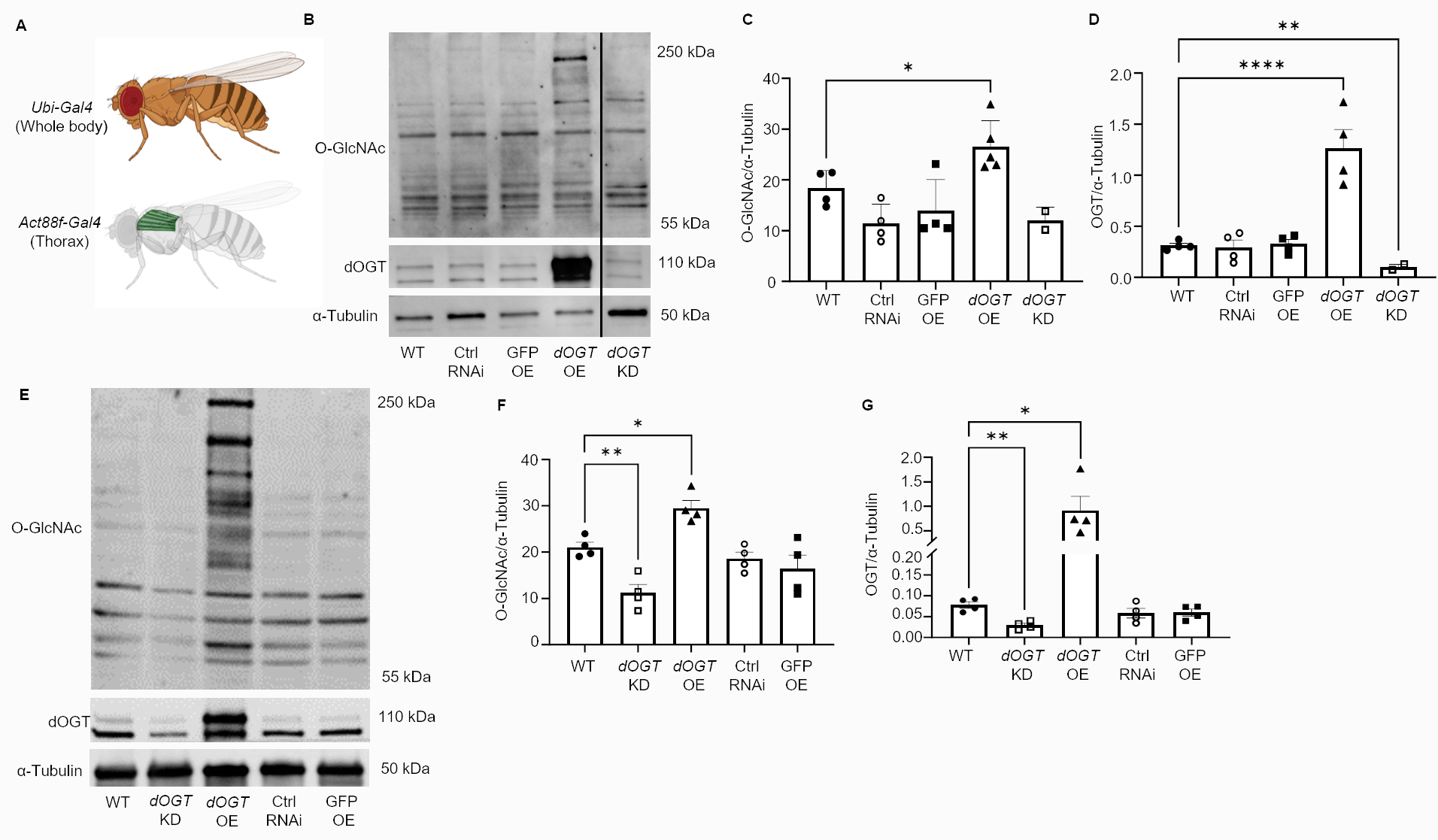

Supplement: Supplementary Figure 2 — Overexpression or knockdown of OGT in the whole body or thorax of Drosophila melanogaster. (A) Schematic of drivers for tissue-specific dOGT knockdown or overexpression used in Drosophila melanogaster experiments. Images were created with Biorender. (B) Representative western blot images of O-GlcNAc, dOGT, and α-Tubulin expression from Ubi-Gal4-driven dOGT KD or OE flies. Black line indicates spliced-together lanes, however, the lanes were run on the same gel, but were noncontiguous. (C–D) Densitometric graphs of the ratio of O-GlcNAc and dOGT to α-Tubulin from Ubi-Gal4-driven dOGT KD or OE flies, n = 2-5. Whole-body dOGT KD hindered growth and viability of fruit flies; therefore, biological replicates were limited. (E) Representative western blot images of O-GlcNAc, dOGT and α-Tubulin expression from Act88f-Gal4-driven dOGT KD or OE flies. (F–G) Densitometric graphs of the ratio of O-GlcNAc and dOGT to α-Tubulin from Act88f-Gal4-driven dOGT KD or OE flies, n = 4. Data from multiple replicates are presented as mean ± SEM, each dot represents independent biological replicate. Statistical analyses were done using the Student’s t test. *p<0.05, **p<0.01, ****p<0.0001. [file Image_2.tif]

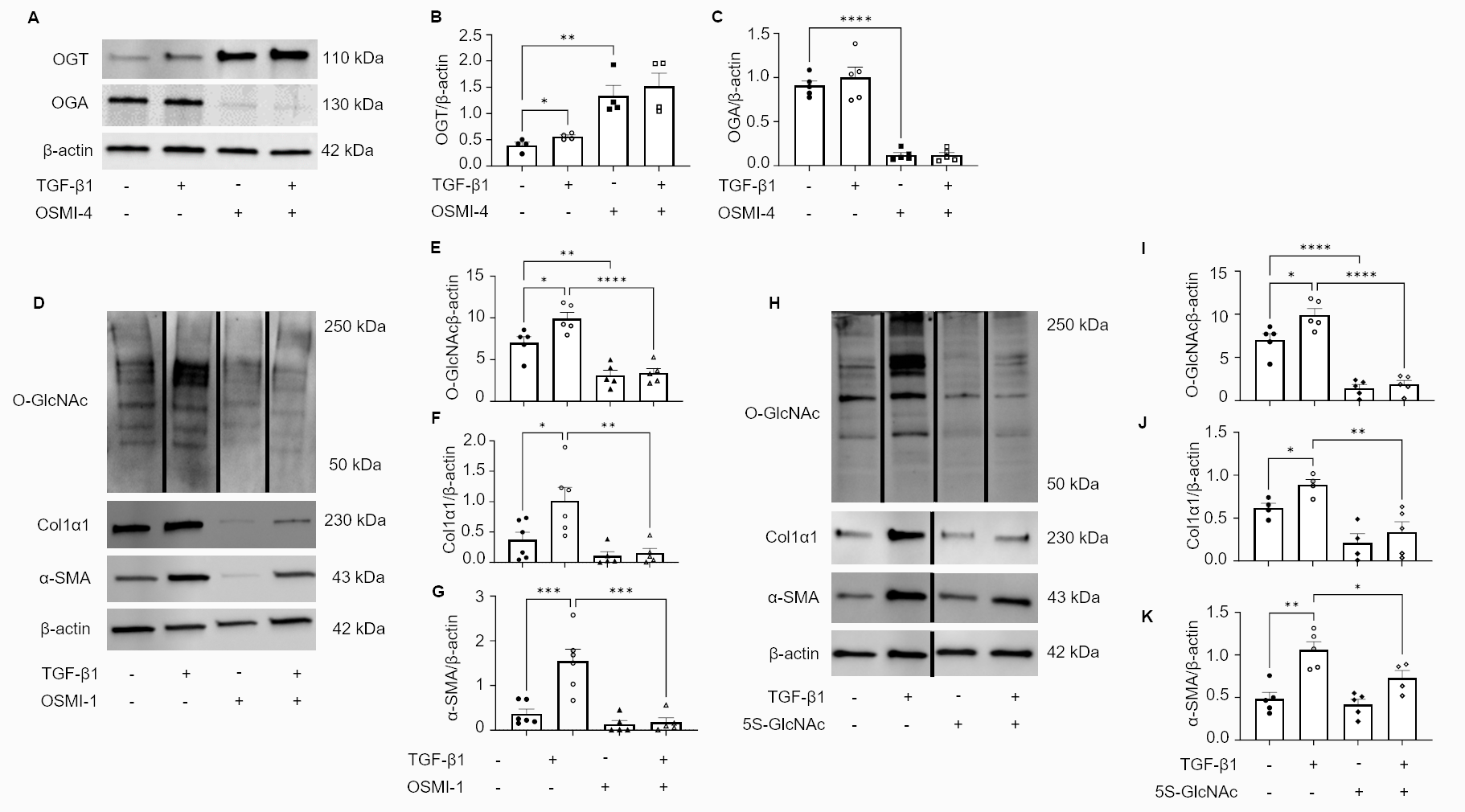

Supplement: Supplementary Figure 3 — OSMI-1 and 5S-GlcNAc, two different OGT inhibitors, attenuate TGF-β1 induction of FMT markers, similar to OSMI-4. (A) Representative western blot images of OGT, OGA, and β-actin expression from HLFs with or without OSMI-4 inhibition (10 µM) followed by TGF-β1 (5 ng/mL) stimulation for 48 hours. (B, C) Densitometric graphs of the ratio of OGT and OGA to β-actin, n = 4-5. (D) Representative western blot images of O-GlcNAc, Col1α1, α-SMA, and β-actin expression from HLFs with or without OSMI-1 inhibition (25 µM) followed by TGF-β1 (5 ng/mL) stimulation for 48 hours. (E–G) Densitometric graphs of the ratio of O-GlcNAc, Col1α1, α-SMA, to β-actin, n = 5-6. (H) Representative western blot images of O-GlcNAc, Col1α1, α-SMA, and β-actin expression from HLFs with or without 5S-GlcNAc inhibition (50 µM) followed by TGF-β1 (5 ng/mL) stimulation for 48 hours. (I–K) Densitometric graphs of the ratio of O-GlcNAc, Col1α1, α-SMA, to β-actin, n = 5-6. Black line indicates spliced-together lanes, however, the lanes were run on the same gel, but were noncontiguous. Data from multiple replicates are presented as mean ± SEM, each dot represents independent biological replicate. Outliers determined by the 1.5 IQR rule were excluded, and statistical analyses were done using the Student’s t-test. *p<0.05, **p<0.01, ***p<0.001, ****p<0.0001. [file Image_3.tif]
